# Mitochondrial Diabetes Is Associated with the *ND4* G11696A Mutation

**DOI:** 10.3390/biom13060907

**Published:** 2023-05-30

**Authors:** Yu Ding, Shunrong Zhang, Qinxian Guo, Jianhang Leng

**Affiliations:** 1 Central Laboratory, Hangzhou First People’s Hospital, Zhejiang University School of Medicine, Hangzhou 310006, China; 2 Department of Geriatrics, Hangzhou First People’s Hospital, Zhejiang University School of Medicine, Hangzhou 310006, China

**Keywords:** T2DM, mtDNA, m.G11696A, tRNA mutations, mitochondrial dysfunctions

## Abstract

Type 2 diabetes mellitus (T2DM) is a common endocrine disorder which remains a large challenge for clinicians. Previous studies have suggested that mitochondrial dysfunction plays an active role in T2DM progression, but a detailed mechanism is still elusive. In the current study, two Han Chinese families with maternally inherited T2DM were evaluated using clinical, genetic, molecular, and biochemical analyses. The mitochondrial genomes were PCR amplified and sequenced. Phylogenetic and bioinformatic analyses were used to assess the potential pathogenicity of mitochondrial DNA (mtDNA) mutations. Interestingly, the matrilineal relatives of these pedigrees exhibited variable severity of T2DM, in particular, the age at onset of T2DM varied from 26 to 65 years, with an average of 49 years. Sequence analysis revealed the presence of *ND4* G11696A mutation, which resulted in the substitution of an isoleucine for valine at amino acid (AA) position 312. Indeed, this mutation was present in homoplasmy only in the maternal lineage, not in other members of these families, as well as 200 controls. Furthermore, the m.C5601T in the tRNA^Ala^ and novel m.T5813C in the tRNA^Cys^, showing high evolutional conservation, may contribute to the phenotypic expression of *ND4* G11696A mutation. In addition, biochemical analysis revealed that cells with *ND4* G11696A mutation exhibited higher levels of reactive oxygen species (ROS) productions than the controls. In contrast, the levels of mitochondrial membrane potential (MMP), ATP, mtDNA copy number (mtDNA-CN), Complex I activity, and NAD^+^/NADH ratio significantly decreased in cell lines carrying the m.G11696A and tRNA mutations, suggesting that these mutations affected the respiratory chain function and led to mitochondrial dysfunction that was involved in T2DM. Thus, our study broadened the clinical phenotypes of m.G11696A mutation.

## 1. Introduction

T2DM is a serious public health problem that is widespread in China, affecting approximately 10% of the adult population [1]. It is a complex metabolic disorder that is characterized by abnormal levels of carbohydrates, fats, and proteins [2]. This disease can be caused by single gene mutations or multi-factorial conditions resulting from interactions between environmental and genetic risk factors [3]. Of inherited factors, a maternal excess in the transmission of T2DM was implicated in many case–control studies [4], suggesting that mutation in mtDNA is the molecular basis for this disorder [5]. mtDNA is maternally inherited and not entirely influenced by recombination events. Moreover, owing to the lack of a DNA repair system, mtDNA has a higher mutation rate than nuclear DNA [6]. In fact, since the landmark discovery of mitochondrial diabetes was the identification of a 10.4 kb large deletion in mtDNA [7] and m.A3243G mutation in tRNA^Leu(UUR)^ [8], a growing number of T2DM-associated mtDNA mutations has been reported, such as tRNA^Ile^ T4291C, tRNA^Glu^ A14692G, and T14709C mutations [9,10,11]. However, to date, the molecular pathogenesis of these mutations in T2DM progression remains unclear.

In this study, we reported two Han Chinese families with T2DM. Through the application of PCR-Sanger sequencing, we identified the existence of *ND4* G11696A mutation in both families. To see the contributions of m.G11696A mutation to T2DM progression, we generated cybrid cells which were derived from six patients with this mutation and four controls without this mutation. In addition, the cellular ATP, MMP, ROS, NAD^+^/NADH ratio, mtDNA-CN, and Complex I activity were determined.

## 2. Materials and Methods

### 2.1. Subjects and Clinical Assessments

**Figure 1:**
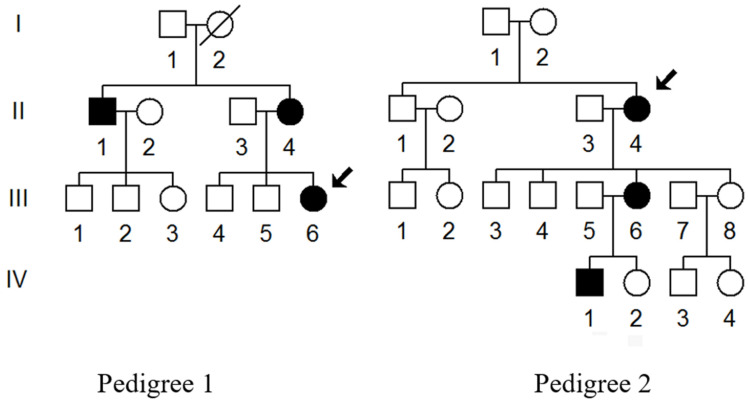
Two Chinese families with T2DM; arrows indicate the probands (III-6 in Pedigree 1 and II-4 in Pedigree 2).

We ascertained two families (Pedigree 1 and Pedigree 2) with T2DM via Hangzhou First People’s Hospital (Figure 1). Members of these pedigrees were evaluated to identify both personal and medical histories of diabetes, deafness, and other clinical abnormalities. Moreover, 200 healthy subjects (100 males and 100 females), aged from 31 to 42, with an average of 38 years, were recruited as controls. This study was approved by the Ethics Committee of the Hangzhou First People’s Hospital (Approval Number: 2021-171-01). Written informed consent, as well as consent to publish these details, was obtained from all participants.

The diagnosis of T2DM was based on the criteria proposed by the American Diabetes Association [12], which are as follows: (1) a fasting plasma glucose (FPG) level ≥7.0 mmol/L; (2) a 2 h plasma glucose level after 75 g oral glucose tolerance test (OGTT) ≥11.1 mmol/L; and (3) the level of Hemoglobin A1c (HbA1c) ≥6.5%. For biochemical assessment, serum FPG was determined using the regular laboratory methods (Beckman Coulter AU5800). In addition, the OGTT was carried out by a measurement of plasma glucose concentrations at 0 and 2 h after the administration of 75 g of glucose.

### 2.2. Screening for the Entire MtDNA Variants

Genomic DNA was extracted from the blood of each participant using Paxgene Blood DNA Isolation kits (QIAGEN, Hilden, Germany). For screening the mtDNA mutations/variants, the affected individuals’ (Pedigree 1: II-1, II-4 and III-6; Pedigree 2: II-4, III-6 and IV-1) DNA fragments spanning the 16,569 bp mitochondrial genome were PCR amplified using 24 primers as described previously [13]. The PCR products were subsequently analyzed using an ABI 3700 automated DNA instrument; the data were compared to the revised Cambridge reference sequences (rCRS) to screen the mutations/variants (GenBank accession number: NC_012920.1) [14].

### 2.3. Haplogroup Classification

Using the nomenclature of mitochondrial haplogroups, we assigned the full mitochondrial sequences of the probands (III-6 in Pedigree 1; II-4 in Pedigree 2) to define mitochondrial haplogroups [15].

### 2.4. Phylogenetic Conservation Analysis

To further assess the pathogenic roles of mtDNA mutations, phylogenetic analysis was performed. In brief, 17 species were selected for this analysis, as described previously [16]. Moreover, the sequence alignment of the mtDNA was performed using the ClustalW program (https://www.ebi.ac.uk/Tools/msa/clustalw2/ (accessed on 10 May 2023) [17].

### 2.5. Population Screening

The frequencies of m.G11696A, m.C5601T, and m.T5813C mutations were further screened in 200 unrelated healthy subjects and all of the members of these two pedigrees. The primers for the amplification of *ND4* gene were forward: 5′-TCA CTC TCA CTG CCC AAG AA-3′, revised: 5′-GGA GAA TGG GGG ATA GGT GT-3′, while the primers for the amplification of tRNA^Cys^ and tRNA^Ala^ genes were forward: 5′-CTA ACC GGC TTT TTG CCC-3′, revised: 5′-ACC TAG AAG GTT GCC TGG CT-3′. After PCR and electrophoresis, the target products were purified and sequenced. The data were then compared with the rCRS (GenBank accession number: NC_012920.1) to detect the occurrence of m.G11696A, m.C5601T, or m.T5813C mutation [14].

### 2.6. Bioinformatics Analyses

To see whether m.C5601T and m.T5813C mutations affected the thermodynamic changes in tRNA^Ala^ and tRNA^Cys^, the online RNA Fold webserver program (http://rna.tbi.univie.ac.at/cgi-bin/RNAfold.cgi (accessed on 10 May 2023) was used to predict the minimum free energy (MFE) of tRNAs with and without these mutations [18]. The wild-type sequence of tRNA^Ala^ was AAG GGC TTA GCT TAA TTA AAG TGG CTG ATT TGC GTT CAG TTG ATG CAG AGG GGG TTT TGC AGT CCT TA; the sequence of tRNA^Ala^ with m.C5601T mutation was AAG GGC TTA GCT TAA TTA AAG TGG CTG ATT TGC GTT CAG TTG ATG CAG AGG GGT TTT TGC AGT CCT TA; the wild-type sequence for tRNA^Cys^ was AGC TCC GAG GTG ATT TTC ATA TTG AAT TGC AAA TTC GAA GAA GCA GCT TCA AAC CTG CCG GGG CTT; and the sequence of tRNA^Cys^ with m.T5813C mutation was AGC TCC GAG GTG ACT TTC ATA TTG AAT TGC AAA TTC GAA GAA GCA GCT TCA AAC CTG CCG GGG CTT.

### 2.7. Qualification of MtDNA-CN

The relative mtDNA-CN was determined by real-time fluorescent quantitative PCR (RT-qPCR), according to our previous study using the 2^−ΔΔCt^ method [19]. The primer sequence for the amplification of the mt-*ND1* was forward: 5′-AAC ATA CCC ATG GCC AAC CT-3′; reverse: 5′-AGC GAA GGG TTG TAG TAG CCC-3′. The primer for the amplification of the β-globin gene was forward: 5′- GAA GAG CCA AGG ACA GGT AC-3′; reverse: 5′-CAA CTT CAT CCA CGT TCA CC-3′. Each experiment was performed in triple.

### 2.8. Cell Lines and Cultured Conditions

Lymphoblastoid cell lines were immortalized by transformation with Epstein–Barr virus (EBV), as described elsewhere [20,21]. Cell lines were derived from six affected matrilineal relatives (Pedigree 1: II-1, II-4 and III-6; Pedigree 2: II-4, III-6 and IV-1) and four controls (Pedigree 1: III-1 and III-2; Pedigree 2: III-1 and III-2). These cell lines were grown in RPMI 1640 medium (Invitrogen Carlsbad, CA, USA) supplemented with 10% FBS.

### 2.9. ATP Measurements

The Cell Titer-Glo^®^ Luminescent Cell Viability Assay kit (Promega, Madison, WI, USA) was used for the measurement of cellular ATP levels, according to the manufacturer’s instructions with some modifications [22]. Briefly, the cells were harvested and resuspended in ultrapure water. The working solution was diluted accordingly and added to the 96-well white plate. Then, ATP releasing buffer was added for ATP extraction, and cells were added to the plate. Finally, the autofluorescence signal was detected by a microplate reader. The results were corrected with cell numbers.

### 2.10. Analysis of MMP

MMP from cybrid cells was examined with JC-10 Assay Kit–Flow Cytometry (Abcam, Cambridge, UK) following the general manufacturer’s recommendations with some modifications [23]. The ratios of fluorescence intensity Ex/Em = 490/590 nm and 490/529 nm (FL_590_/FL_529_) were recorded to delineate the MMP level of each sample. The relative ratios of the FL_590_/FL_529_ geometric mean between mutant and control cell lines were calculated to represent the level of MMP.

### 2.11. Determining the ROS Production

To analyze the ROS level, a total of 2 × 10^6^ cells were first incubated with the fluorescent probe 2,7-dichlorodihydrofluorescein for 30 min, after which the cells were analyzed using a fluorescence plate reader, as described previously [24].

### 2.12. Analysis of NAD^+^/NADH Ratio

The NAD^+^/NADH ratio was measured using the WST-8 NAD^+^/NADH Assay Kit (Beyotime, Shanghai, China), as recommended by the manufacturer’s instructions. Absorbance was measured at 450 nm on a microplate reader (ELX800, BioTek, Winooski, VT, USA) [25,26]. The NAD^+^/NADH ratio was calculated as follows: [NAD_total_ − NADH]/NADH.

### 2.13. Determining the Complex I Activity

For analyzing Complex I specific activity, the mitochondrion was first isolated from cybrid cell lines on ice using different centrifugations, as suggested previously [27]. Complex I activity was determined with 10 μg/mL antimycin A and 2 mm KCN by following a decrease in the absorbance due to the NADH oxidation at 340 nm in assay buffer, as detailed elsewhere [28].

### 2.14. Statistical Analysis

Student’s *t*-test was used to assess the statistical significance between unpaired samples. All analyses were performed using SPSS software version 20.0. We regarded the *p* < 0.05 as statistically significant.

## 3. Results

### 3.1. Clinical Features of Two Chinese Families with T2DM

We enrolled two families with maternally inherited T2DM via Hangzhou First People’s Hospital (Figure 1). In Pedigree 1, the proband (III-6) was a 40-year-old woman who came to the Hangzhou First People’s Hospital for the regular treatment of T2DM; she was administrated with metformin and insulin therapy (0.4 μ/kg). Her comprehensive personal and medical history revealed that she developed T2DM when she was 38. Her family history suggested that her matrilineal relatives (II-1 and II-4) were also diabetic carriers. Moreover, the proband’s grandmother (I-2) died from diabetes several years ago.

In Pedigree 2, the proband (II-4) was a 70-year-old woman who also lived in Hangzhou city of Zhejiang Province. She started to suffer from T2DM when she was 65. The HbA1c, FGP, as well as OGTT results strongly indicated that she was a diabetic carrier. Furthermore, among 11 matrilineal relatives, 3 of them developed DM at different ages (Table 1). Moreover, these members showed no other clinical abnormalities, including cancer, hearing loss, or neurological or infection disorders.

### 3.2. Screening for Mitochondrial Mutations

**Figure 2:**
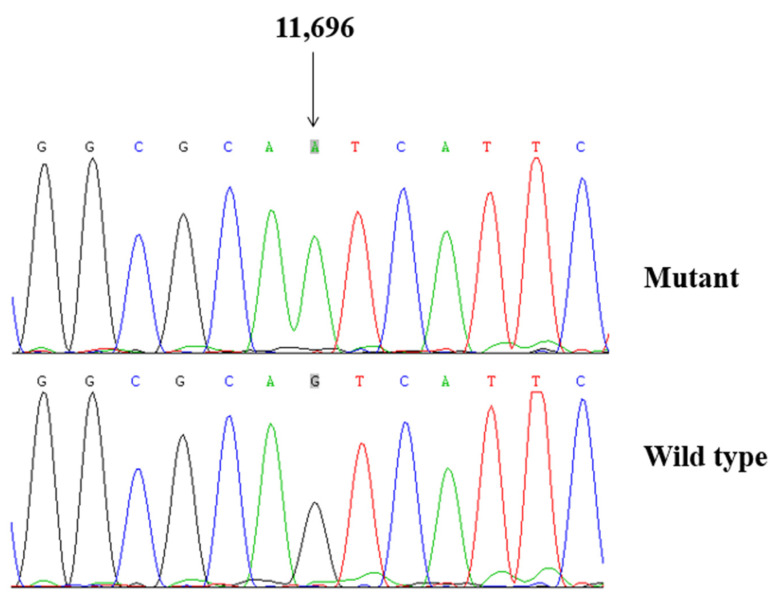
Identification of *ND4* G11696A mutation by Sanger sequencing.

**Figure 3:**
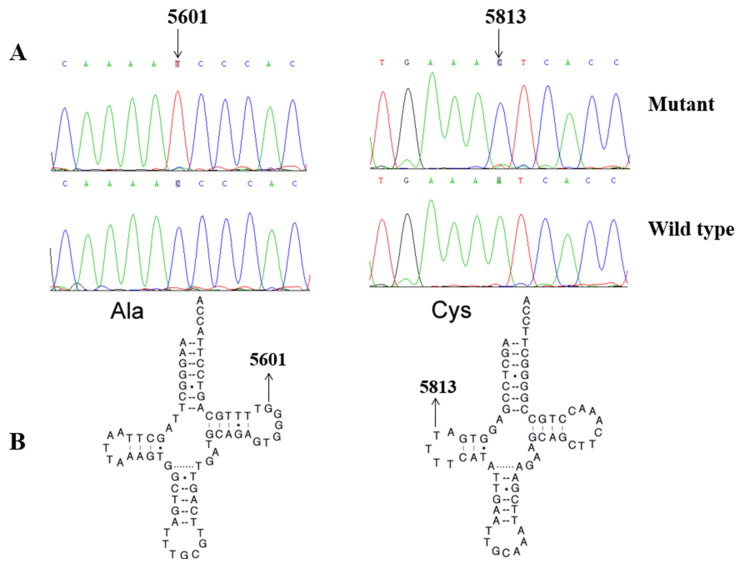
(**A**). Identification of tRNA^Ala^ C5601T and tRNA^Cys^ T5813C mutations by Sanger sequencing. (**B**) The locations of m.C5601T in tRNA^Ala^ and m.T5813C in tRNA^Cys^.

To explore the molecular basis of T2DM, we screened the mtDNA mutations of affected matrilineal relatives (Pedigree 1: II-1, II-4 and III-6; Pedigree 2: II-4, III-6 and IV-1) using PCR and direct sequencing analysis. As shown in Table 2, a comparison of the resultant sequences with rCRS led us to identify 60 variants in the mitochondrial genome which belonged to Eastern Asian mitochondrial haplogroup D4 and D4a, respectively [15]. There were 11 variants in D-loop, 3 variants in 12S rRNA, 3 variants in 16S rRNA, and 2 mutations in tRNAs (m.C5601T and m.T5813C), while the rest of the variants were located as OXPHOS-related genes. In addition, 13 missense mutations were identified, including *ND1* C3497T (Ala to Val), *ND2* A4833G (Thr to Ala) and C5178A (Leu to Met), *A8* C8414T (Leu to Phe), *A6* A8701G (Thr to Ala) and A8860G (Thr to Ala), *ND3* A10398G (Thr to Ala), *ND4* G11696A (Val to Ile) and A12026G (Ile to Val), *ND5* G13928C (Ser to Thr), *Cyt b* C14766T (Thr to Ile), A15326G (Thr to Ala), and A15851G (Ile to Val). These variants in RNAs and polypeptides were further evaluated using phylogenetic analysis and sequences from other 16 vertebrates, including mouse [29], bovine [30], and *Xenopus laevis* [31]. We noticed that except for the *ND4* G11696A, tRNA^Ala^ C5601T, and tRNA^Cys^ T5813C mutations (Figure 2 and Figure 3), others were not well conserved, suggesting that they may be involved in the pathogenesis of T2DM (Table 3).

### 3.3. Population Screening

To see the potential pathogenicity of *ND4* G11696A, tRNA^Ala^ C5601T, and tRNA^Cys^ T5813C mutations, we screened the frequencies of these mutations in all members of 2 pedigrees, as well as 200 healthy controls using PCR-Sanger sequencing. We noticed that the m.G11696A, m.C5601T, and m.T5813C mutations were only presented in the matrilineal relatives of two pedigrees, but they were absent in the control subjects.

### 3.4. M.C5601T and m.T5813C Mutations Altered tRNAs Secondary Structures

**Figure 4:**
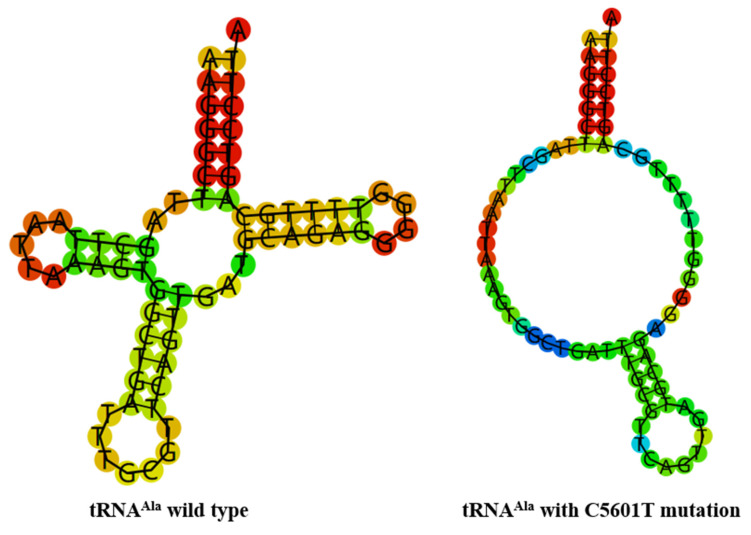
Predict the secondary structure of tRNA^Ala^ with and without m.C5601T mutation.

**Figure 5:**
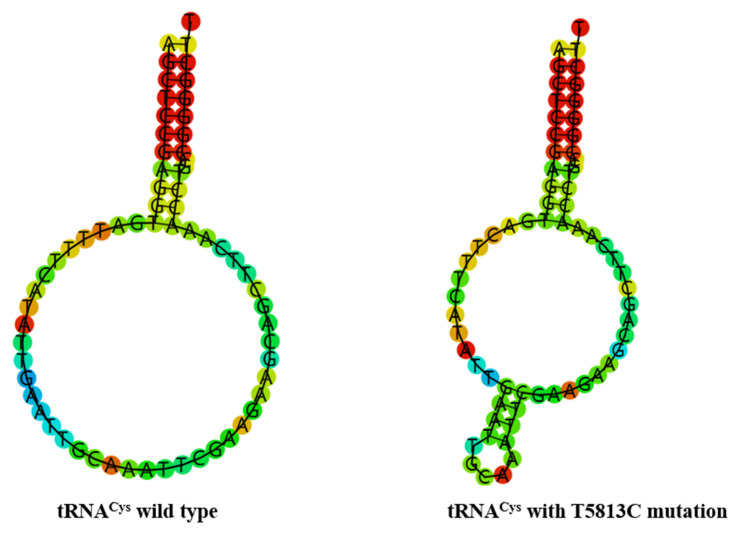
Predict the secondary structure of tRNA^Cys^ with and without m.T5813C mutation.

To see whether m.C5601T and m.T5813C mutations influenced the tRNA structure, the online RNA Fold webserver (http://rna.tbi.univie.ac.at/cgi-bin/RNAfold.cgi (accessed on 10 May 2023) was used to assess the thermodynamic changes in tRNAs with and without these mutations. As shown in Figure 4 and Figure 5, we found that these mutations affected the secondary structures of corresponding tRNAs, strongly indicating that they may be pathogenic.

### 3.5. MtDNA-CN Decreased

**Figure 6:**
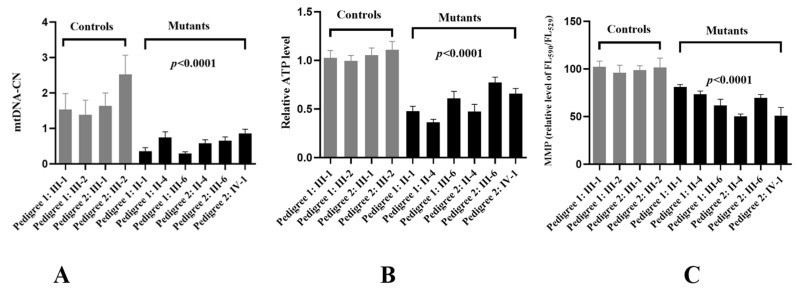
Analysis of mitochondrial functions in cybrids. (**A**) Relative levels of mtDNA-CN. (**B**) ATP determination. (**C**) MMP analysis.

MtDNA-CN was considered as a proxy for mitochondrial function [35]. To see whether m.G11696A mutation affected mtDNA function, RT-qPCR was performed to determine the mtDNA-CN. As shown in Figure 6A, a ~37% average reduction was identified in individuals who carried the m.G11696A mutation when compared with the controls (*p* < 0.0001).

### 3.6. Reduced in ATP Production

To see whether m.G11696A mutation affected mitochondrial functions, we generated cybrid cell lines derived from six patients and four controls. As shown in Figure 6B, an approximately 33.6% average drop in ATP was observed in mutant cell lines with the m.G11696A mutation when compared with the controls (*p* < 0.0001).

### 3.7. MMP Analysis

MMP generated by proton pumps (Complexes I, III, and IV) was an essential component in the process of energy storage during oxidative phosphorylation (OXPHOS) [36]. The MMP levels in cell lines were measured using a fluorescence probe JC-10 assay via flow cytometry. As shown in Figure 6C, subjects with m.G11696A mutation exhibited about a 23.5% decrease in MMP levels compared with the controls (*p* < 0.0001).

### 3.8. The Enhanced in ROS Levels

**Figure 7:**
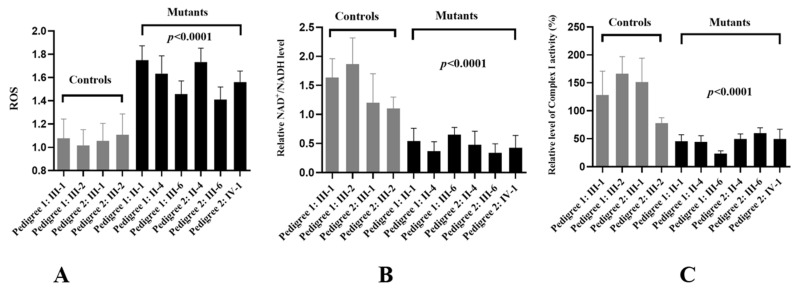
Mitochondrial dysfunction in mutant cells. (**A**) Analysis of ROS levels in cybrids; (**B**) determining the NAD^+^/NADH ratio between two groups; (**C**) complex I activity analysis.

Mitochondrial ROS have been gradually recognized as important signaling mediators in a wide range of cellular processes, such as metabolic adaptation, adaptive responses to hypoxia, cellular differentiation, and autophagy [37]. The ROS levels of cybrid cells were measured using a fluorescence plate reader, as shown in Figure 7A. The levels of ROS generation in the mutant cell lines harboring the G11696A mutation were much higher than the controls without this mutation (*p* < 0.0001).

### 3.9. NAD^+^/NADH Ratio Decreased

The NAD^+^/NADH ratio was a key regulator of the cells’ metabolism and believed to influence oxidative stress (OS) [38]. We found that, compared with the controls, patients with m.G11696A mutation exhibited much lower levels of the NAD^+^/NADH ratio (Figure 7B).

### 3.10. Reduced Activity of Complex I

To see the effects of m.G11696A mutation on OXPHOS function, we analyzed the activity of respiratory Complex I in ten cybrid cells. As shown in Figure 7C, we noticed that Complex I was markedly decreased in cells with m.G11696A mutation, as compared with the controls (*p* < 0.0001).

## 4. Discussion

In this study, we performed clinical, genetic, molecular, and biochemical characterizations of two genetic unrelated Chinese pedigrees with maternally inherited T2DM. Notably, T2DM was only presented in the matrilineal lineage of these pedigrees, strongly indicating that mutations in mtDNA were the important contributors to this disease. In fact, the most important role of mitochondria was generating ATP for the maintenance of cellular process [39]. Mitochondria also played an essential role in ROS-mediated signaling transduction, apoptosis, and hormone biogenesis [40]. Herein, we determined that *ND4* G11696A mutation was associated with T2DM in two pedigrees. Indeed, the A-to-g transition at 11,696 resulted in the substitution of an isoleucine for valine at amino acid position 312 [41]. Notably, the valine at position 312 in *ND4* protein was located in a predicted transmembrane region, 28 AAs aminoterminal to the R340H Leber’s hereditary optic neuropathy (LHON) mutation [42]. A previous study revealed a severe Complex I deficiency in muscle biopsy carrying this mutation, highlighting the importance of m.G11696A to mitochondrial dysfunction [43]. In addition, the m.G11696A mutation was regarded to be a risk factor for mitochondrial disorders [41,44,45,46,47,48] (Table 4).

Interestingly, the m.C5601T mutation in tRNA^Ala^ and m.T5813C mutation in tRNA^Cys^ was also detected in our study (Figure 2 and Figure 3). At the molecular level, the m.C5601T mutation resided at the extremely conserved nucleotide of tRNA^Ala^ (position 59); the mutation at that position was involved in the biochemical and molecular interactions between the TψC loop and D-arm [49]. Importantly, the m.C5601T mutation created a novel Watson–Crick base-pairing (55T-59C) [32,33,34]. Furthermore, the novel m.T5813C mutation occurred at position 14 in the D-arm of tRNA^Cys^; intriguingly, the m.T7501C mutation, which was located at the same position of tRNA^Ser(UCN)^, was found to be associated with cardiovascular diseases [50,51]. Thus, we speculated that the m.T5813C mutation, which was similar to the m.T7501C mutation, may also have a pathological consequence for T2DM. Moreover, bioinformatics analysis revealed that the m.C5601T and m.T5813C mutations affected the secondary structures of tRNA^Ala^ and tRNA^Cys^, respectively (Figure 4 and Figure 5). Therefore, m.C5601T and m.T5813C mutations may cause failure in tRNAs metabolism and lead to mitochondrial dysfunction which was responsible for T2DM.

MtDNA-CN was a mitochondrial function marker that reflected its depletion, energy reserves, and OS [52]. Recent studies demonstrated that lower mtDNA content was found to be associated with insulin resistance, T2DM, and impair pancreatic β-cell functions [53,54,55], which was consistent with our results.

Trans-mitochondrial technology was frequently used to evaluate the contribution of mtDNA mutations to the OXPHOS function since noise from the nuclear genetic background was adjusted [56]. In this study, we used this technology to test mitochondrial functions in cybrids carrying m.G11696A mutation. We found that, compared with control cell lines, mutant cybrids had much lower levels of ATP, MMP, NAD^+^/NADH ratio, and Complex I activity. In fact, the reduction in ATP production in m.G11696A cells was likely a consequence of the decrease in the proton electrochemical potential gradient of mutant mitochondria [57]. MMP was a major component of proton motive force, which played an essential role in mitochondrial bioenergetics, metabolic, and signaling functions [58,59]. Loss of MMP was a critical event in deciding cell fate [60].

NAD^+^ and NADH were the most important redox pairs in the cell. In particular, NAD^+^, a key co-enzyme in cellular energy metabolism, could be adaptive response to OS and affect mitochondrial function [61]. The NAD^+^/NADH ratio set the intracellular redox environment, controlled antioxidant capacity, and regulated cell signaling [62]. Mutant cells showed a marked decreased in their ratio indicated that m.G11696A exhibited more OS and mitochondrial dysfunction than the controls.

The cell lines with m.G11696A showed significant reductions in Complex I activity, as in the case of T2DM-associated *ND6* T14502C mutation [63]. Complex I was a large protein complex responsible for acquiring electrons by oxidizing NADH to NAD^+^ and transferring these electrons to CoQ [64]. It was also a primary site where electrons leaked into the mitochondrial matrix and bound with molecular oxygen to form superoxide [65]. The deficient activity of Complex I caused by m.G11696A mutation would increase the production of ROS [66]. Excess ROS can increase mitochondrial dysfunction, protein damage, lipid peroxidation, and impair antioxidant functions [67]. Thus promoting pancreatic β-cell death or apoptosis and contributing to T2DM progression [68].

In conclusion, our study suggested that m.G11696A mutation was associated with T2DM. Moreover, tRNA^Ala^ C5601T and tRNA^Cys^ T5813C mutations may alter the structure and tRNA functions, subsequently leading to failure in tRNAs metabolism, and they play as synergistic role in the phenotypic expression of m.G11696A mutation. Thus, our study broadened the clinical presentations of m.G11696A mutation. The main limitation of the current study was the relatively small sample size. Further studies including more T2DM samples are needed to verify this conclusion.

## Figures and Tables

**Table 1:** Clinical and biochemical features of several members of two Chinese families with T2DM.

Subjects	Gender	Age at Test (years)	Age at Onset (years)	Fasting Glucose (mmol/L)	Oral Glucose Tolerance (mmol/L)	HbA1c (%)
Pedigree 1 (II-1)	Female	65	55	7.0	14.55	6.9
Pedigree 1 (II-4)	Male	68	60	5.9	12.5	8.5
Pedigree 1 (III-6)	Male	40	38	5.3	11.2	6.5
Pedigree 2 (II-4)	Male	70	65	7.5	13.8	6.7
Pedigree 2 (III-6)	Male	50	49	6.8	12.7	7.0
Pedigree 2 (IV-1)	Female	26	26	6.5	11.8	6.6
Pedigree 2 (III-5)	Female	52	/	5.3	10.2	5.5

**Table 2:** mtDNA sequence variants in two families with T2DM.

Gene	Position	Mutation (Amino Acid Change)	Conservation (H/B/M/X) ^a^	rCRS ^b^	Pedigree 1	Pedigree 2	Previously Reported ^c^
D-loop	73	A to G		A	G	G	Yes
	195	T to C		T		C	Yes
	215	A to G		A	G		Yes
	263	A to G		A	G	G	Yes
	310	T to CTC		T	CTC		Yes
	489	T to C		T	C	C	Yes
	495	C to T		C		T	Yes
	16,189	T to C		T		C	Yes
	16,223	C to T		C	T	T	Yes
	16,362	T to C		T		C	Yes
	16,519	T to C		T	C		Yes
12S rRNA	709	G to A	G/G/A/-	G		A	Yes
	750	A to G	A/A/A/-	A	G	G	Yes
	1438	A to G	A/A/A/G	A	G	G	Yes
16S rRNA	1719	G to A	G/A/A/T	G		A	Yes
	2706	A to G	A/G/A/A	A	G	G	Yes
	3107	delN		N	delN	delN	Yes
ND1	3396	C to T		C	T		Yes
	3497	C to T (Ala to Val)	A/A/L/S	C		T	Yes
	3970	C to T		C	T	T	Yes
ND2	4706	A to G		A	G		Yes
	4833	A to G (Thr to Ala)	T/I/I/L	A		G	Yes
	4883	C to T		C	T	T	Yes
	5178	C to A (Leu to Met)	L/T/T/T	C	T		Yes
tRNA^Ala^	5601	C to T	C/C/C/C	C	T		Yes
tRNA^Cys^	5813	T to C	A/A/A/A	A		C	No
CO1	6086	T to C		T		C	Yes
	6968	C to T		C	T		Yes
	7028	C to T		C	T	T	Yes
CO2	7621	T to C		T		C	Yes
	7768	A to G		A		G	Yes
	8200	T to C		T	C		Yes
A8	8414	C to T (Leu to Phe)	L/F/M/W	C	T	T	Yes
	8512	A to G		A		G	Yes
A6	8701	A to G (Thr to Ala)	T/S/L/Q	A	G	G	Yes
	8860	A to G (Thr to Ala)	T/A/A/T	A	G	G	Yes
	9004	C to T		C	T		Yes
CO3	9540	T to C		T	C	C	Yes
	9950	T to C		T	C		Yes
ND3	10,310	G to A		G	A		Yes
	10,398	A to G (Thr to Ala)	T/T/T/A	A	G	G	Yes
	10,400	C to T		C	T	T	Yes
ND4	10,873	T to C		T	C	C	Yes
	11,696	G to A (Val to Ile)	V/T/T/M	G	A	A	Yes
	11,719	G to A		G	A	A	Yes
	11,944	T to C		T		C	Yes
	12,026	A to G (Ile to Val)	I/I/M/L	A	G		Yes
ND5	12,373	G to A		G		A	Yes
	12,705	C to T		C	T	T	Yes
	13,269	A to G		A	G		Yes
	13,928	G to C (Ser to Thr)	S/S/V/V	G		C	Yes
ND6	14,569	G to A		G		A	Yes
	14,668	C to T		C	T	T	Yes
Cyt b	14,766	C to T (Thr to Ile)	T/I/S/S	C	T	T	Yes
	15,043	G to A		G	A	A	Yes
	15,301	G to A		G	A	A	Yes
	15,326	A to G(Thr to Ala)	T/M/I/I	A	G	G	Yes
	15,670	T to C		T		C	Yes
	15,784	T to C		T	C		Yes
	15,851	A to G (Ile to Val)	I/A/S/M	A		G	Yes

^a^ Conservation of amino acid for polypeptides or nucleotide for RNAs in human (H), bovine (B), mouse (M), and *Xenopus laevis* (X). ^b^ rCRS: revised Cambridge Reference Sequences. ^c^ Please see Mitomap database (www.mitomap.org (accessed on 10 May 2023)).

**Table 3:** Molecular characterization of m.C5601T and m.T5813C mutations identified in this study.

tRNA Species	Nucleotide Change	Number of Nucleotides in tRNA	CI (%)	Location in tRNA	Disease Association	References
tRNA^Ala^	C5601T	59	100	TψC loop	EH, NSHL, LHON, T2DM	[32,33,34]
tRNA^Cys^	T5813C	14	100	D-arm	T2DM	This study

Abbreviations: CI, conservation index; EH, essential hypertension; NSHL; non-syndromic hearing loss; LHON, Leber’s hereditary optic neuropathy; T2DM, type 2 diabetes mellitus.

**Table 4:** Overview of *ND4* G11696A mutation.

Number	Age (years)	Gender	Clinical Feature	Family History	References
1	17	Male	LHON	Yes	[41]
2	15	Female	LHON	Yes	[41]
3	19	Female	LHON	Yes	[41]
4	8	Female	LHON	Yes	[41]
5	38	Female	LHON	Yes	[41]
6	72	Female	LHON	Yes	[44]
7	36	Male	EH	Yes	[45]
8	Unknown	Male	Idiopathic oligoasthenospermia	No	[46]
9	21	Male	LHON	Yes	[47]
10	18	Male	LHON	Yes	[47]
11	20	Male	LHON	Yes	[47]
12	18	Male	LHON	Yes	[47]
13	12	Male	LHON	Yes	[47]
14	25	Male	LHON	Yes	[47]
15	35	Male	LHON	Yes	[47]
16	10	Male	LHON	Yes	[47]
17	17	Female	NSHL	Yes	[48]

Abbreviations: LHON, Leber’s hereditary optic neuropathy; EH: essential hypertension; NSHL: non-syndromic hearing loss.

## Data Availability

The datasets for this study will be available from the corresponding authors upon reasonable request.
